# In Steatotic Cells, ATP-Citrate Lyase mRNA Is Efficiently Translated through a Cap-Independent Mechanism, Contributing to the Stimulation of De Novo Lipogenesis

**DOI:** 10.3390/ijms21041206

**Published:** 2020-02-11

**Authors:** Luisa Siculella, Laura Giannotti, Mariangela Testini, Gabriele V. Gnoni, Fabrizio Damiano

**Affiliations:** 1 Laboratory of Molecular Biology, Department of Biological and Environmental Sciences and Technologies, University of Salento, 73100 Lecce, Italy; laura.giannotti@unisalento.it (L.G.); mariangelates@gmail.com (M.T.); fabrizio.damiano@unisalento.it (F.D.); 2 Laboratory of Biochemistry, Department of Biological and Environmental Sciences and Technologies, University of Salento, 73100 Lecce, Italy; gabriele.gnoni@unisalento.it

**Keywords:** ATP-citrate lyase, Cap-independent translation, de novo lipogenesis, endoplasmic reticulum stress, internal ribosome entry site, lipid droplets, non-alcoholic fatty liver diseases

## Abstract

Non-alcoholic fatty liver disease (NAFLD) is a chronic disease in which excessive amount of lipids is accumulated as droplets in hepatocytes. Recently, cumulative evidences suggested that a sustained de novo lipogenesis can play an important role in NAFLD. Dysregulated expression of lipogenic genes, including ATP-citrate lyase (ACLY), has been found in liver diseases associated with lipid accumulation. ACLY is a ubiquitous cytosolic enzyme positioned at the intersection of nutrients catabolism and cholesterol and fatty acid biosyntheses. In the present study, the molecular mechanism of ACLY expression in a cell model of steatosis has been reported. We identified an internal ribosome entry site (IRES) in the 5′ untranslated region of the ACLY mRNA, that can support an efficient mRNA translation through a Cap-independent mechanism. In steatotic HepG2 cells, ACLY expression was up-regulated through IRES-mediated translation. Since it has been demonstrated that lipid accumulation in cells induces endoplasmic reticulum (ER) stress, the involvement of this cellular pathway in the translational regulation of ACLY has been also evaluated. Our results showed that ACLY expression was increased in ER-stressed cells, through IRES-mediated translation of ACLY mRNA. A potential role of the Cap-independent translation of ACLY in NAFLD has been discussed.

## 1. Introduction

Non-alcoholic fatty liver disease (NAFLD) represents a spectrum of hepatic disorders, ranging from simple fatty liver (non-alcoholic fatty liver, NAFL) to non-alcoholic steatohepatitis (NASH), characterized by inflammation and hepatocytic injury, i.e., cell degeneration and necroapoptosis [1]. In NAFLD, triglycerides are accumulated and stored in the cytoplasm of hepatocytes in the form of lipid droplets, giving origin mainly to macrovesicular steatosis. This pathology is closely associated with obesity, type 2 diabetes, and insulin resistance (IR).

Dietary chylomicron remnants and lipolysis from adipose tissue are the major sources of fatty acids for lipid accumulation in NAFLD. For a long time, hepatic de novo lipogenesis (DNL) has not been considered as a relevant cause of steatosis. The main reason was that, in healthy humans, only 5–10% of the hepatic triglycerides derives from the DNL, being the majority of fatty acids originating from dietary sources and peripheral lipolysis [2].

In recent years, increasing evidences show that, in liver, DNL is up-regulated in individuals with hepatic insulin resistance and/or NAFLD [3,4]. The molecular mechanisms leading to the induction of DNL and consequently to hepatic steatosis are complex and poorly understood [3,5]. Previous reports have suggested that endoplasmic reticulum (ER) stress plays a crucial role in lipid metabolism [6,7,8]. ER stress occurs when ER protein-folding capacity is altered, leading to the activation of an evolutionarily conserved unfolded protein response (UPR) pathway in order to restore ER homeostasis. It has been observed that, together with other cellular processes, UPR pathway induces lipid biosynthesis and plays a fundamental role in the development of NAFLD [6,7,8]. Furthermore, it has been reported that lipid accumulation itself induces ER stress [9], creating the condition of a vicious circle, in which ER stress determined by lipid accumulation induces the synthesis of further lipids [9].

To study the molecular mechanism of hepatic lipid accumulation, animal models with either genetic or nutritional induction of NAFLD have been used [10]. Human HepG2 cells cultured in a medium supplemented with different amount of free fatty acids (FFAs) have been used as an in vitro model of steatosis [9,11]. FFA supplementation to the cultured hepatocytes triggers the intracellular accumulation of lipid droplets, thus emulating hepatic steatosis. In this model, palmitic (C16:0) and oleic (C18:1) acids are the main FFAs used to induce lipid droplets accumulation, because they are the most abundant fatty acids in hepatic triglycerides both in healthy subjects and in patients with NAFLD [11].

By using this model, the regulation of Sterol Regulatory Element-Binding Protein-1 (SREBP-1) transcription factor, the “master regulator” of lipogenic genes [12], has been investigated [9].

SREBP-1 is synthesized as inactive precursor (pSREBP-1) bound to the endoplasmic reticulum (ER), where its regulatory domain co-localizes with an ER-embedded protein, SREBP-cleavage-activating protein (SCAP). SCAP functions as a sensor of membrane cholesterol levels. When cells become depleted in cholesterol, the SREBP–SCAP complex translocates from the ER to the Golgi, where a two-step proteolytic cleavage releases the nuclear and active form of SREBP-1 (nSREBP-1), allowing its entry into the nucleus [12].

Following accumulation of lipid droplets in cells, an increase of the expression of SREBP-1 and of SREBP-1 target genes, such as fatty acid synthase (FASN) and acetyl-CoA carboxylase 1 (ACACA), has been observed [9].

ATP-citrate lyase (ACLY) is a ubiquitous cytosolic enzyme positioned at the intersection of nutrient catabolism and fatty acid and cholesterol biosyntheses. In mammals, ACLY is highly expressed in lipogenic tissues, such as adipose tissue, liver, and lactating mammary glands [13]. ACLY catalyzes the conversion of citrate and coenzyme A (CoA) into acetyl-CoA and oxaloacetate, coupled with the hydrolysis of ATP. Citrate is synthesized in mitochondria during Krebs cycle, through the condensation of acetyl-CoA with oxaloacetate. In a good energetic state, citrate is transported from mitochondria into the cytosol by mitochondrial citrate carrier [14]. Cytosolic acetyl-CoA, generated by ACLY, is then used as a starter molecule for the synthesis of fatty acids and cholesterol, as well as of several important lipidic molecules including isoprenoids, coenzyme Q10, and dolichol [15].

ACLY is activated by different kinases-mediated phosphorylation [16] and by acetylation [17], whereas transcriptional induction of *Acly* gene is mediated by SREBP-1 [18].

The role of ACLY in the development of hepatic steatosis has been poorly studied so far. In this study we investigated the regulation of ACLY expression in an in vitro model of hepatic steatosis, represented by HepG2 cells treated with a mixture of palmitic and oleic fatty acids. We showed that accumulation of lipids stimulates the expression of ACLY in HepG2 cells at translational level, through an internal ribosome entry site (IRES) located in the 5′ untranslated region (5′ UTR) of ACLY mRNA. Moreover, ACLY IRES supports the expression of this enzyme upon induction of ER stress in HepG2 cells treated with an ER stressor, such as tunicamycin or thapsigargin.

Our data demonstrated that up-regulation of ACLY expression in steatotic HepG2 occurs at the translational level. The role of the Cap-independent translation of ACLY mRNA in NAFLD has been discussed.

## 2. Material and Methods

### 2.1. Cell Culture

HepG2 cells were maintained in Dulbecco’s modified Eagle’s medium (DMEM) (Corning Life Sciences, Tewksbury, MA, USA) supplemented with 10% (*v*/*v*) heat-inactivated fetal bovine serum (FBS) (Corning Life Sciences), penicillin G (100 units/mL), and streptomycin (100 μg/mL) (Corning Life Sciences). Cells were kept at 37 °C in a humidified atmosphere containing 5% CO_2_. In order to induce lipid accumulation in cells, HepG2 cells were plated at a density of 1 × 10^6^ cells into 25 cm^2^ flask and incubated for 48 h. Then, a mixture of free oleic and palmitic fatty acids (FFAs) (Sigma-Aldrich, Milano, Italy) in the molar ratio of 2:1 was added to the culture medium at the final concentrations of 0.75 mM for the indicated times [9]. The mixture (10 mM) of FFAs was prepared by dissolving the fatty acids in fatty acid-free bovine serum albumin (BSA) (Sigma-Aldrich, Milano, Italy). The molar ratio of FFAs to BSA was 5:1. Induction of ER stress was performed incubating HepG2 cells in culture medium supplemented with 1 μg/mL tunicamycin or 300 nM thapsigargin (Sigma-Aldrich, Milano, Italy) for the indicated times [19].

### 2.2. Synthesis of Mono- and Di-Cistronic Constructs

The 5′ UTR of the human ACLY mRNA (GenBank^®^ accession number NM_198830.1) was amplified from total RNA by RT-PCR, using the primers listed in Table 1. The identity of the amplimers was checked by DNA sequencing [20]. The ACLY 5′ UTR amplimer was then digested with HindIII and NcoI and inserted into the pGL3prom vector (Promega Italia, Milano, Italy) to obtain pGL3-ACLY. The plasmids pRF, phpRF, have been described previously [20]. The ACLY 5′ UTR amplimer was digested with EcoRI and NcoI and inserted either into the intercistronic region of pRF or pHpRF, obtaining the dicistronic constructs pR-ACLY-F or pHpR-ACLY-F, respectively. Promoterless dicistronic construct pR-ACLY-F(-P) was made by removing the SV40 (simian virus 40) promoter sequence from pR-ACLY-F [20]. The promotorless pRF(-P) has been previously described [20].

### 2.3. Transient Transfection Assay

HepG2 cells were transiently transfected with luciferase reporter construct as previously described [19], using Metafectene^®^ Pro (Biontex Laboratories, München, Germany) transfection reagent. After cells lysis, *Renilla* luciferase (RL) and firefly luciferase (FL) activities were measured using the Dual Luciferase Reporter Assay System (Promega Italia, Milano, Italy). To evaluate the effect of lipid droplets accumulation or ER stress on ACLY IRES activity, cells were transfected with pR-ACLY-F. After 24 h, cells were incubated with 0.75 mM FFAs or with ER stressor (1 μg/mL tunicamycin or 300 nM thapsigargin) for the indicated times. For the transfection normalization, the pcDNA3.1/His/lacZ plasmid, coding for β-galactosidase, was used. The β-galactosidase activity was determined using a β-galactosidase assay. Differences in the β-galactosidase activity measured in control and in treated-cells were not statistically significant, confirming that the experimental conditions did not influence the β-galactosidase expression.

### 2.4. Isolation of RNA and qRT-PCR Analysis

Total RNA extraction from cultured cells and qRT-PCR analysis were carried out as described previously [21]. Quantitative gene expression analysis was performed (CFX Connect™ Real-Time PCR Detection System, BioRad Laboratories, Milano, Italy) using SYBR Green technology (FluoCycle, Euroclone, Milano Italy) and 18S rRNA for normalization. The sequence of primers used for the quantification of ACLY and SREBP-1 mRNA are reported in Table 1.

RT-PCR was also performed to rule out cryptic splicing within the intercistronic region in the dicistronic mRNA [19]. cDNA was synthesized by using total RNA extracted from control un-transfected cells or cells transfected with pRF or pR-ACLY-F. Then, PCR reaction was carried out by using cDNA as template and the primers CSFor-CSRev reported in Table 1.

### 2.5. Immunoelectrophoretic Analysis

Western blot analysis was carried out as reported previously [22]. After electrophoretic transfer of proteins to nitrocellulose, the blots were probed with antibody directed against ACLY or SREBP-1 (Santa Cruz Biotechnology Inc., Dalla, TX, USA). The detection system employed was the ECL Pico Plus (Thermo Fisher, Milano, Italy).

### 2.6. ACLY Half-Life Analysis

HepG2 cells were cultured at a density of 1 × 10^6^ cells into 25 cm^2^ flask and incubated for 48 h. To investigate the influence of lipid droplets accumulation or ER stress on ACLY stability, cells were incubated in culture medium supplemented with 0.75 mM FFAs, or with ER stressor (1 μg/mL tunicamycin or 300 nM thapsigargin). Then, 2 μg/mL puromycin (Sigma-Aldrich, Milano, Italy), inhibitor of protein synthesis, was added to the medium and cells were incubated for the indicated times. At different times, cells from each flask were harvested and Western blotting analysis was performed as described above. Autoradiograms were quantified by densitometric scanning.

### 2.7. Statistical Analysis

Data represent means ± standard deviation (S.D.) of 3–5 replicates. Statistical analysis was performed using ANOVA with Tukey’s post-test (GraphPad Software, Inc., San Diego, CA, USA). *p*-value < 0.05 was considered to achieve statistical significance.

## 3. Results

### 3.1. ACLY Expression Changes in HepG2 Cells upon Lipid Accumulation.

To investigate the effect of lipid droplets accumulation on ACLY expression, HepG2 cells were cultured in a medium supplemented with 0.75 mM FFAs up to 48 h. The concentration of FFAs (0.75 mM) is in the range of plasmatic FFAs levels in patients with NAFLD [9,23]. This treatment did not affect cell viability (data not shown). Concentrations greater than 0.75 mM have not been tested because of their cytotoxic effect [9].

**Figure 1:**
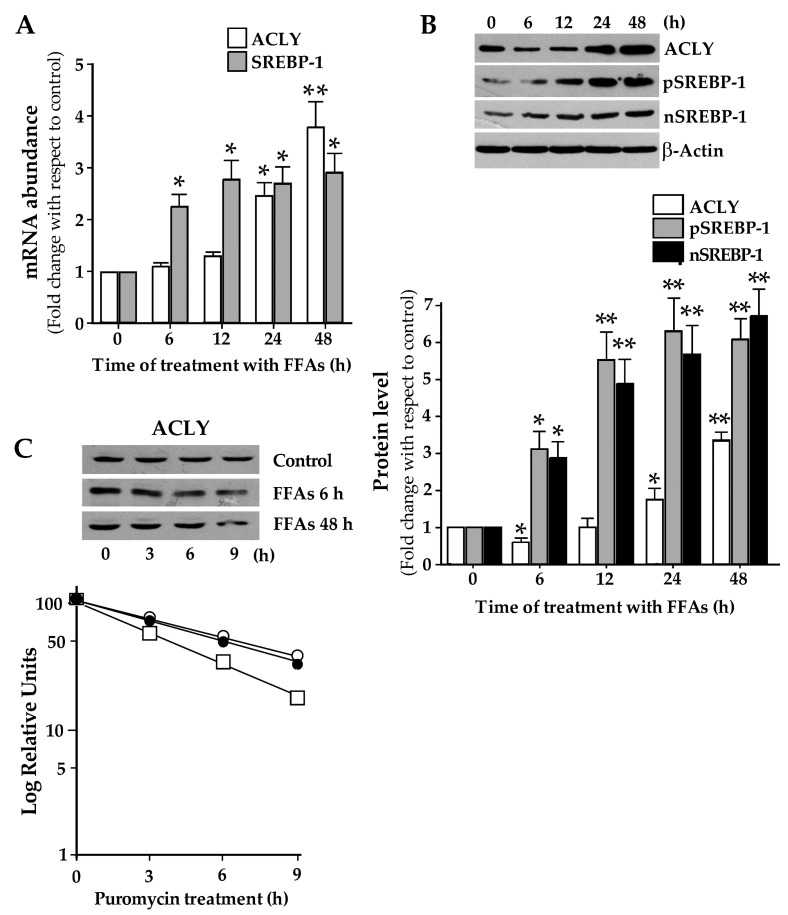
Free fatty acids (FFAs) treatment induces the expression of ATP-citrate lyase (ACLY). (**A**) HepG2 cells were incubated in the presence of a mixture of free oleic and palmitic fatty acids (FFAs) in the molar ratio of 2:1 for the indicated times. Cells were then harvested, and total RNA was extracted. ACLY and Sterol Regulatory Element-Binding Protein-1 (SREBP-1) mRNA levels were determined by using RT-qPCR and normalized with 18S rRNA. Values were reported in histograms as fold change relative to the untreated control cells. Values are means ± S.D., *n* = 5 (For ACLY 0 h vs. 24 h * *p* ≤ 0.05; 0 h vs. 48 h ** *p* ≤ 0.01; For SREBP-1, 0 h vs. each time of treatment, * *p* ≤ 0.05). (**B**) Proteins (60 μg) were prepared from FFAs-treated cells, separated by SDS/PAGE and immunoblotted with antibody against ACLY or SREBP-1. The content of ACLY, precursor SREBP-1 (pSREBP-1) and mature SREBP-1 (nSREBP-1) in FFAs-treated cells was analyzed by Western blotting, quantified by densitometric analysis, and expressed as fold change relative to untreated control cells. Values are means ± S.D., *n* = 4 (For ACLY, 0 h vs. 6 h and 24 h * *p* ≤ 0.05; 0 h vs. 48 h ** *p* ≤ 0.01; For pSREBP-1 and nSREBP-1, 0 h vs. 6 h * *p* ≤ 0.05; 0 h vs. 12 h, or 24 h, or 48 h ** *p* ≤ 0.01). (**C**) HepG2 cells, incubated for 6h or 48h in DMEM with either vehicle (BSA) or 0.75 mM FFAs, were then treated with 2 μg/mL puromycin, inhibitor of protein synthesis. At different times, cells were harvested and the content of ACLY protein was measured by Western blot analysis. The semilog plot represents the decay curve of ACLY protein in control (filled circle), 6 h FFAs-treated (open square), and 48h FFAs-treated (open circle) HepG2 cells. The results are from a representative experiment, with similar results being obtained in three independent experiments.

Results of RT-qPCR showed that, in the first 12 h of FFAs treatment, ACLY mRNA levels remained unchanged, while they increased by about 4-fold after 48 h (Figure 1A). As shown in Figure 1B, incubation of HepG2 with FFAs led to a reduction in the level of ACLY protein at 6 h of treatment. Then ACLY levels showed a constant increase, becoming about 3.5-fold greater than the control after 48 h of treatment (Figure 1B). In agreement with previous results [9], treatment with FFAs caused an increment of SREBP-1 mRNA abundance (Figure 1A). The content of precursor (pSREBP-1) and active nuclear (nSREBP-1) of SREBP-1 protein increased in FFAs-treated cells with respect to control (Figure 1B).

To explain the biphasic trend of ACLY protein levels observed in HepG2 cells upon incubation with FFAs, ACLY protein stability was examined in FFAs-supplemented and in control cells. Results showed that ACLY half-life measured in cells treated with FFAs for 6h was reduced (∼3.8 h) when compared with control cells (∼5.3 h). Conversely, the apparent half-life of ACLY protein increased upon incubation of cells with FFAs for 48h (∼6.8 h FFAs) (Figure 1C).

### 3.2. Functional Characterization of 5′ Untraslated Region of ACLY mRNA

We questioned whether translational regulation of ACLY could be involved in the increment of ACLY protein content in steatotic HepG2 cells, previously reported for SREBP-1a [9].

**Figure 2:**
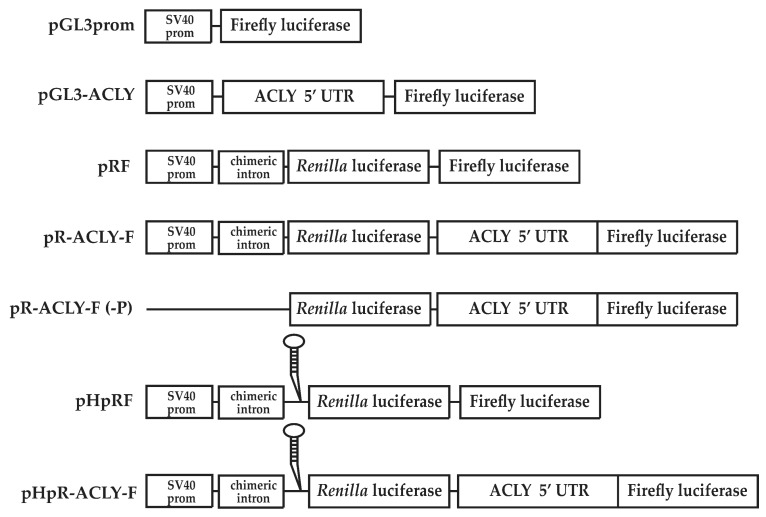
Constructs in pGL3prom, pRF and pHpRF. pGL3-ACLY contains ACLY 5′ untranslated region (UTR) inserted into pGL3prom upstream firefly luciferase (FL) cistron. The control dicistronic vector pRF contains the SV40 early promoter (prom), a chimeric intron, and the cDNAs encoding *Renilla* luciferase (RL) and FL separated by a short linker sequence. pR-ACLY-F construct is obtained by cloning the ACLY 5′ UTR between the two cistrons RL and FL. The ACLY 5′ UTR was also cloned between the two cistrons RL and FL of pHpRF obtaining the pHpR-ACLY-F construct. The pHpRF differs from pRF because it contains an inverted repeat upstream the first cistron, in order to form a stem–loop at the 5′ end of the transcript. pRF(-P) and pR-ACLY-F(-P) constructs are identical to their corresponding pRF constructs described above, except that they do not contain the SV40 early promoter and the chimeric intron.

**Figure 3:**
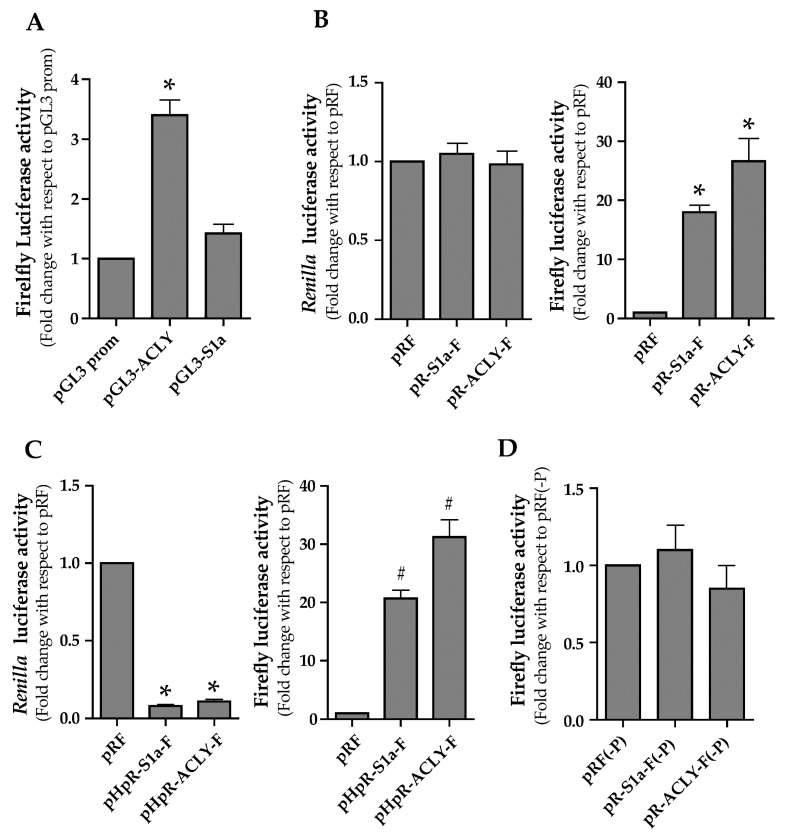
Evaluation of a putative internal ribosome entry site (IRES) in the 5′ UTR of ACLY mRNA. (**A**) HepG2 cells were co-transfected with pGL3prom, pGL3-ACLY or pGL3S1a constructs together with the control plasmid pcDNA3.1/HisB/lacZ. At 24 h after transfection, FL activity was measured and normalized with respect to β-galactosidase activity. Values are means ± S.D. (*n* = 6) and are reported as a percentage of FL activity determined in cells transfected with the empty vector pGL3 prom. (pGL3 prom vs. pGL3-ACLY *p ≤ 0.05). (**B**) HepG2 cells were co-transfected with pR-S1a-F or pR-ACLY-F constructs, together with pcDNA3.1/HisB/lacZ used for the normalization of transfection efficiency. RL and FL activities, normalized to β-galactosidase activity, are reported as the fold change relative to those determined in cells transfected with the control vector pRF. Values are means ± S.D., (*n* = 6). (pRF vs. pR-S1a-F and pR-ACLY-F * *p* ≤ 0.01). (**C**) HepG2 cells were co-transfected with pRF, pHpR-S1a-F or pHpR-ACLY-F, together with pcDNA3.1/HisB/lacZ used for the normalization of transfection efficiency. RL and FL activities, normalized to the β-galactosidase activity, were reported as the fold change relative to those determined in cells transfected with the control vector pRF. Values are means ± S.D., (*n* = 6). (Right panel, pRF vs. pHpR-S1a-F and pHpR-ACLY-F * *p* ≤ 0.05; left panel, pRF vs. pHpR-S1a-F and pHpR-ACLY-F ^#^ *p* ≤ 0.01). (**D**) The promoterless pRF(-P) or pR-ACLY-F(-P) constructs were co-transfected together with pcDNA3.1/HisB/lacZ into HepG2 cells. At 24 h after transfection, cells were harvested for determination of FL activity, which was normalized to the β-galactosidase activity. Values were reported as the fold change relative to the FL activity measured in HepG2 cells transfected with pRF(-P). Values are means ± S.D., (*n* = 6).

To investigate this aspect, the 5′ UTR cDNA of human ACLY mRNA (163 bp) was inserted upstream the firefly luciferase (FL) cistron of pGL3prom plasmid to create the pGL3-ACLY (Figure 2). The pGL3-S1a construct containing a 185 bp fragment of human SREBP-1a 5′ UTR was used as a positive control [9,20]. Transfection with pGL3-ACLY resulted in FL activity 3.3-fold higher than that produced by control pGL3prom (Figure 3A). In cells transfected with pGL3-S1a, FL activity was 1.4-fold higher (Figure 3A) than that produced from control cells transfected with the empty vector pGL3prom.

We hypothesized that an internal ribosome entry site (IRES)-dependent mechanism could be implicated in the regulation of ACLY expression. To test this hypothesis, the cDNA corresponding to the 5′ UTR of ACLY mRNA was inserted into the dicistronic vector pRF [24]. pRF plasmid contains two reporter genes: the first cistron (*Renilla* luciferase, RL) is under the control of the SV40 promoter and is translated via Cap-dependent mechanism, whereas the second FL cistron is translated independently from the Cap structure [24]. The 5′ ACLY 5′ UTR cDNA was cloned upstream the FL cistron to obtain pR-ACLY-F (Figure 2). When pR-ACLY-F was transfected in HepG2 cells, FL activity was about 26-fold higher than that measured with the control pRF (Figure 3B). In cells transfected with the positive control pR-S1a-F construct, FL activity was approximately 20-fold higher when compared to the pRF control plasmid (Figure 3B). The presence of the ACLY 5′ UTR between the two reporter genes did not alter RL activity (Figure 3B). Thus, these results support the hypothesis that the leader region of ACLY mRNA could contain a putative IRES.

Different mechanisms alternative to IRES, such as ribosomal reinitiation at the FL start codon, or generation of monocistronic FL mRNA by splicing or by transcription from a cryptic promoter, could also explain the enhanced activity of FL observed in pR-ACLY-F transfected cells.

To rule out the hypothesis of a ribosomal reinitiation mechanism, the ACLY 5′ UTR was cloned into the pHpRF vector, to obtain the pHpR-ACLY-F construct (Figure 2). The rationale for using such construct was to generate a dicistronic RNA containing, upstream the RL coding region, a stable hairpin structure (-55 kcal/mol), able to reduce the Cap-dependent translation of the RL cistron [24]. Conversely, the Cap-independent translation of the downstream FL cistron should not be affected. Compared to cells transfected with pRF, RL activity was reduced by about 75% (Figure 3C) whereas FL activity increased (Figure 3C) in HepG2 cells transfected with pHpR-ACLY-F. Similar results were obtained in luciferase assay performed with the positive control pHpR-S1a-F construct (Figure 3C).

**Figure 4:**
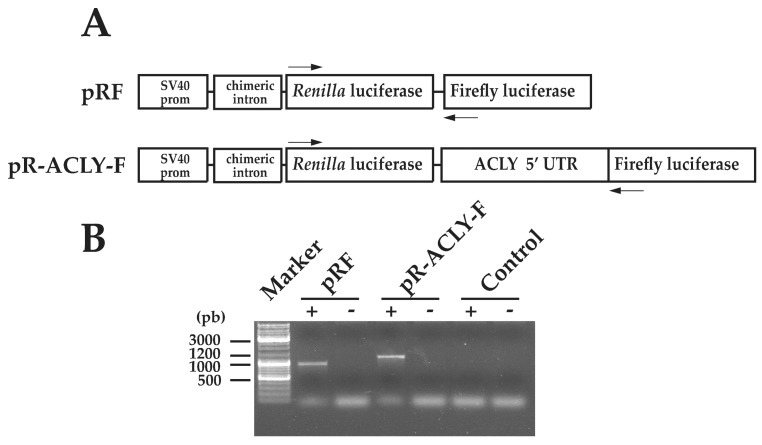
Cryptic splicing does not occur in the dicistronic transcript containing ACLY 5′ UTR. (**A**) Dicistronic constructs used to transfect HepG2 cells are depicted. The arrows indicate the positions of the primers CSFor and CSRev used for RT-PCR and reported in Table 1. RT-PCR analysis was performed as additional test to rule out cryptic splicing within the ACLY 5′ UTR in the intercistronic region. (**B**) RNA was extracted by HepG2 cells transfected with the dicistronic constructs and used as a template for RT-PCR. The amplimers containing the RL and the intercistronic region were analyzed on ethidium-bromide-stained agarose gel. The result is from a representative experiment (*n* = 3).

Upon transfection with the promoter-less pR-ACLY-F (-P) (Figure 2), a low FL activity was measured, ruling out the presence of a cryptic promoter in the ACLY 5′ UTR (Figure 3D). Moreover, RT-PCR was performed by using a couple of primers complementary to the 5′ end of RL and FL genes (Figure 4A). Result showed that a full-length amplimer corresponding to the FL gene was obtained, ruling out the possibility of cryptic splicing (Figure 4B).

### 3.3. Endoplasmic Reticulum-Stress Promotes the ACLY Expression

**Figure 5:**
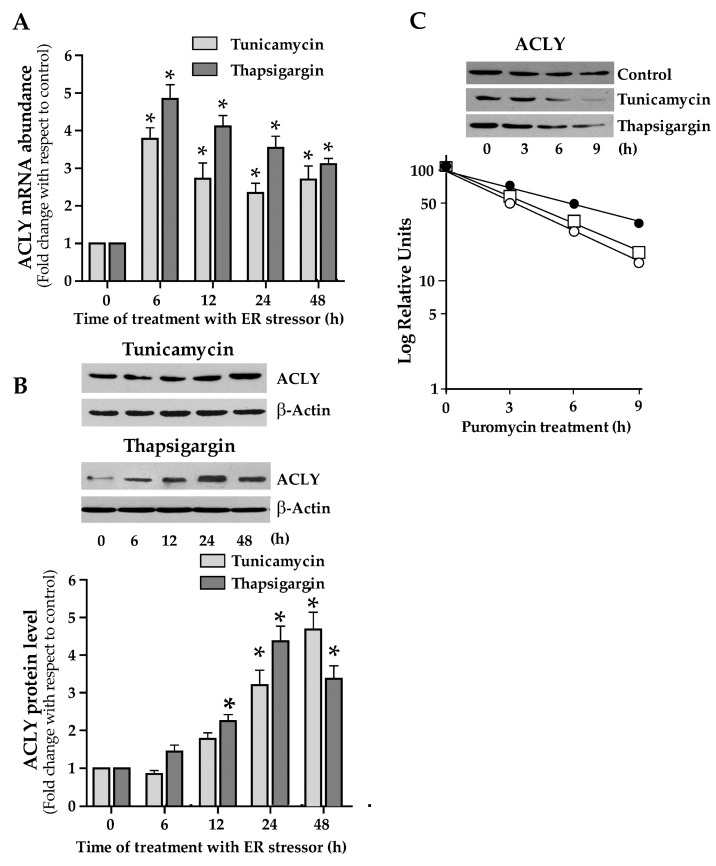
ACLY expression in endoplasmic reticulum (ER)-stressed cells. (**A**) HepG2 cells were incubated in the presence of 1 μg/mL tunicamycin or 300 nM thapsigargin for the indicated times. Cells were then harvested, and total RNA was extracted. ACLY mRNA levels, normalized with 18S rRNA, were reported in histograms as fold change relative to the untreated control cells. Values are means ± S.D., *n* = 5. (0 h vs. 6 h, 12 h, 24 h, and 48 h * *p* ≤ 0.05). (**B**) Proteins (60 μg) were prepared from ER-stressed cells, separated by SDS/PAGE and immunoblotted with the antibody against ACLY. The content of ACLY was analyzed by Western blotting, quantified by densitometric analysis, and was expressed as fold change relative to ACLY content in untreated control cells. Values are means ± S.D., *n* = 4 (0 h vs. 24 h and 48 h, * *p* ≤ 0.05). (**C**) HepG2 cells, incubated in DMEM with 1 μg/mL tunicamycin or 300 nM thapsigargin for 24h, were then treated with 2 μg/mL puromycin. At different times, cells were harvested and the content of ACLY protein was measured by Western blot analysis. The semilog plot represents the decay curve of ACLY protein in control (filled circle), in tunicamycin- (open circle) and thapsigargin-treated (open square) HepG2 cells. The results are from a representative experiment, with similar results being obtained in three independent experiments.

Cap-independent translation mediated by IRES plays an important role in gene expression in cells undergoing to ER stress [25]. For this reason, we investigated the expression of ACLY in HepG2 cells upon ER stress triggered through tunicamycin or thapsigargin addition to the culture medium. In cells treated with 1 µg/mL tunicamycin or 300 nM thapsigargin, ACLY mRNA abundance showed a rapid and consistent increase after 6h of treatment. Then, ACLY mRNA level was reduced, remaining however at values higher than control (Figure 5A). Treatment of cells with tunicamycin or thapsigargin resulted in an increase of ACLY protein levels (Figure 5B). The turnover of ACLY was also investigated in HepG2 cells under ER stress and, when compared to control cells, the half-life of ACLY decreased in ER-stressed cells. Thus, the augmented ACLY level observed in ER-stressed cells cannot be explained by an increment of its stability (Figure 5C).

### 3.4. ER Stress and Lipid Droplets Accumulation Drive ACLY IRES Activity

**Figure 6:**
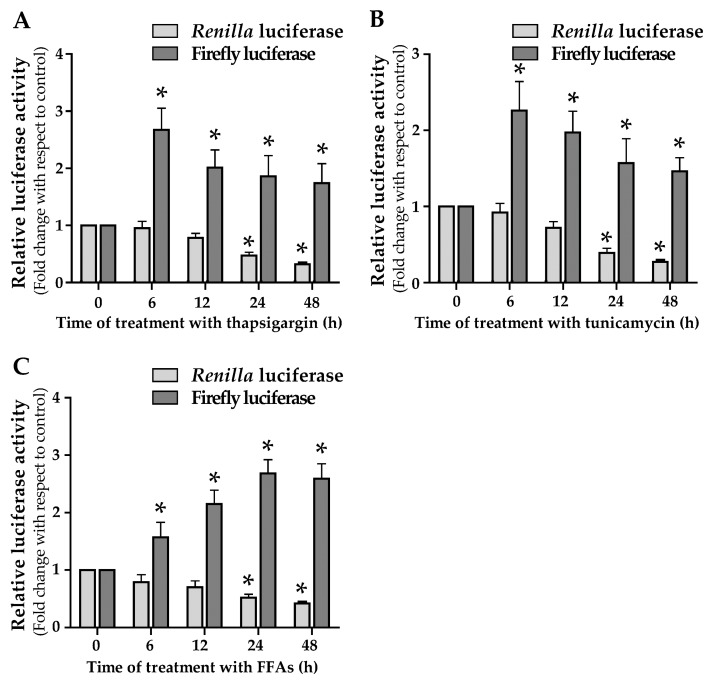
IRES activity is stimulated in ER-stressed cells or in cells under treatment with FFAs. (**A**,**B**) HepG2 cells were co-transfected with the reporter plasmid pR-ACLY-F and with the control plasmid pCMV-SPORT-β-gal. After 24 h, cells were treated with thapsigargin (**A**) or tunicamycin (**B**) for the indicated times and then RL and FL luciferase activities were measured. For each sample, values were reported in histograms as fold change with respect to control, represented by untreated transfected cells. Values are means ± S.D., *n* = 6 (0 h vs. 6 h, 12 h, 24 h, and 48 h * *p* ≤ 0.05). (**C**) HepG2 cells were transiently transfected with pR-ACLY-F. After transfection, cells were incubated in the presence or in the absence of 0.75 mM FFAs. Luciferase activity measured in cells treated with FFAs is reported as fold change with respect to that determined in untreated transfected cells. Values are means ± S.D., *n* = 6 (0 h vs. 6 h, 12 h, 24 h, and 48 h * *p* ≤ 0.05).

The role of ACLY IRES in the induction of ACLY expression in cells under ER-stress has been investigated. For this aim, HepG2 cells were transfected with pR-ACLY-F and then treated with the ER stressors. Treatment with 1 µg/mL tunicamycin or 300 nM thapsigargin caused a time-dependent reduction of RL activity. In contrast, an increase in FL activity was observed in ER stressed cells (Figure 6A,B). Analysis carried out by RT-qPCR showed that the level of RL or FL mRNA abundance was not affected by ER stressors treatment (data not shown). Thus, the increased activity of ACLY IRES observed under stress conditions explains the increase in ACLY protein levels reported in Figure 5B.

In a previous work, we observed that the accumulation of triglycerides in HepG2 cells causes the onset of an ER stress condition, as evidenced by the increased expression of ER stress markers [9]. This observation led us to evaluate the activity of ACLY IRES following lipid droplets accumulation in HepG2. The results here reported show that RL and FL measured in FFAs-treated HepG2 cells change with a trend similar to that observed in ER stressors-treated cells (Figure 6C).

## 4. Discussion

There is growing interest in NAFLD, a disease affecting both adults and children. When not properly treated, NAFLD can cause a series of more serious liver diseases, from steatohepatitis to degeneration of the hepatic parenchyma with fibrosis and cirrhosis, and finally hepatocarcinoma.

Among the factors contributing to the accumulation of lipids in hepatocytes, DNL has long been considered irrelevant. In recent years, this consideration has been questioned, recognizing this metabolic pathway plays a causative role in the hepatic steatosis. Multiple lines of evidence have indicated that dysregulated lipogenesis considerably contributes to NAFLD [3,4,9,26,27].

The molecular mechanism of DNL activation, in a context of large availability of fatty acids as in NAFLD, is largely unknown. IR has been recognized as a fundamental factor contributing to the onset of NAFLD.

In the liver, IR causes an abnormal response in which elevated blood insulin levels don’t effectively suppress gluconeogenesis but potently activate DNL [28,29,30]. In IR liver, activation of DNL, together with increased influx of fatty acids from adipose tissue lipolysis, leads to the accumulation of triglycerides and of lipotoxic metabolites (long-chain acyl-CoAs, diacylglycerol, lysophosphatidic acid, ceramides) [31], thus perpetuating IR [32].

ACLY plays a crucial metabolic role by controlling the flow of glucose carbons to cytosolic acetyl-CoA, the precursor of fatty acids and, then, of triglycerides synthesis. Therefore, ACLY links cellular glucose catabolism to lipid biosynthesis. In mice, it has been observed that dysregulation of ACLY is correlated to hepatic steatosis and IR [33]. In human, the amount of ACLY mRNA is higher in NAFLD patients than in healthy subjects [34]. Silencing of *Acly* gene by RNA interference markedly inhibits the expression of the entire lipogenic pathway in liver of obese mice [33].

In this work, we deeply investigated the molecular mechanism underlying dysregulation of ACLY in NAFLD. This study has been carried out in a cellular model of hepatic steatosis [9,11]. Incubation of HepG2 cells in the presence of a mix of FFAs containing 2:1 oleate and palmitate causes lipid accumulation in the cytosol similar to that observed in steatotic liver [9,11]. All the experiments have been carried out with oleate/palmitate at the concentration of 0.75 mM, which is similar to that of plasmatic non-esterified fatty acids (NEFA) measured in patients with metabolic syndrome [35].

The results reported in this work showed that accumulation of lipids in HepG2 cells led to an increase in ACLY expression (Figure 1). Previously, it has been reported that transcription of *Acly* gene is promoted by an epigenetic mechanism in NAFLD [34]. However, ACLY is a target gene of SREBP-1 transcription factor [18], which is overexpressed in steatotic cells (Figure 1) [9]. Thus, increment of ACLY mRNA abundance in NAFLD can be explained through the transcriptional activation of *Acly* gene by SREBP-1.

In FFAs-treated cells, ACLY protein level changed with a biphasic trend (Figure 1B). After the initial reduction observed at 6h, ACLY level increased up to 48h, reaching values higher than those obtained in the control. The initial decrease of ACLY can be explained by the reduction of its stability (Figure 1C). At 48 h of FFAs treatment, the turnover of ACLY remained unchanged when compared to the control (Figure 1C).

To deepen the molecular mechanism underlying the ACLY trend in steatotic HepG2 cells, we hypothesized the existence of a regulation of ACLY expression at translational level. Indeed, luciferase assays led to the identification of an IRES in the 5′ UTR of ACLY mRNA (Figure 3).

IRESs, elements located in the leader region of a restricted class of mRNA, allow the initiation of mRNA translation independently on the Cap-structure [25,36,37]. The alternative mechanism of translation mediated by IRESs is important when the attenuation of global protein synthesis occurs in cells undergoing stress conditions, such as ER stress, hypoxia, UV irradiation, etc. Notably, ER stress and the sub-consequent activation of the UPR pathway often occur in lipid metabolic disorders, including NAFLD, and are correlated with an enhanced lipogenesis and accumulation of lipids [6,9,38,39]. Results of experiments carried out by using an ER stress inducer, such as tunicamycin or thapsigargin, confirmed that ER-stress induction triggers the ACLY expression at both mRNA and protein level (Figure 5). The finding that ACLY IRES was activated by ER stress induction supports the evidence for an efficient translation of ACLY mRNA through the Cap-independent mechanism (Figure 6). Thus, these data confirm the role of IRES, identified in ACLY 5′ UTR, in promoting ACLY mRNA translation in cells under ER stress.

We hypothesized that activation of IRES-mediated translation of ACLY mRNA could be the mechanism explaining the increased ACLY protein level observed in HepG2 cells upon FFAs treatment (Figure 1). Moreover, in FFAs-treated HepG2 cells luciferase assays demonstrated that activity of ACLY IRES increased in a time-dependent manner (Figure 6C).

In previous studies, IRES-mediated translation has been described for SREBP-1 transcription factor, the master regulator of lipogenic genes [20], and for acetyl-CoA carboxylase 1 [19]. These observations suggest that Cap-independent translation may relate to other lipogenic genes, thus providing a possible explanation of de novo lipogenesis activation in ER-stressed cells and/or in steatosis as well. In addition to the evidence of a high expression of ACLY in NAFLD patients [34], the involvement of IRES dependent translation of endogenous ACLY mRNA in steatotic liver should be demonstrated through in vivo studies.

IRES-mediated Cap-independent translation is often supported by IRES trans-activating factors (ITAFs) [36]. In recent studies, it has been shown an increase of the SREBP-1a expression dependent on binding of hnRNP A1 on SREBP-1a 5′ UTR [9,40]. Therefore, an important question concerns the role of putative ITAFs in the regulation of ACLY expression in steatotic liver.

What is the role of ACLY activation in NAFLD? Increased activity of ACLY and synthesis of acetyl-CoA can support cellular processes other than lipid synthesis. Acetyl-CoA formed by ACLY is also used for protein acetylation. Notably, acetylation of different proteins, including transcription factors and kinase proteins, promotes the synthesis of lipids and, at the same time, inhibits their catabolism [41,42]. The deacetylation of the same proteins by sirtuins has the opposite effect [41,42]. In ER-stressed cells, it has been observed an increase of lysine acetylation of several ER-resident chaperones and enzymes involved in protein post-translational modification to restore a correct folding [43,44]. Acetylated proteins are also involved in the degradation of unfolded protein aggregates (ERAD) as well as in autophagy [43]. Moreover, lipid droplets have been proposed as a temporary depot for proteins destined for degradation by ERAD [45]. Therefore, considering the role of ACLY in both lipogenesis and protein acetylation, the increase of ACLY expression observed in ER-stressed cells could indicate an ACLY function in maintaining the proteostatic balance in the cell.

ACLY has been considered an effective target for the treatment of diseases associated to disorders of lipid metabolism [13,33,46,47]. Thus, the knowledge of molecular mechanism of ACLY regulation at translational level, reported in the present study, provides new elements for therapeutic treatment of metabolic diseases.

## Figures and Tables

**Table 1:** Oligonucleotides used for qRT-PCR analysis, for construction of monocistronic and dicistronic vectors, and for the evaluation of cryptic splicing.

Primer	Oligonucleotide Sequence
ACLY-RTFor	5′-TACATCTGCAAAGTGAAGTGG-3′
ACLY-RTRev	5′-TTCAGCAAGGTCAGCTTCAG-3′
SREBP-1For	5′-ACACCATGGGGAAGCACAC-3′
SREBP-1Rev	5′-CTTCACTCTCAATGCGCC-3′
rRNA18SFor	5′-GTTGGTTTTCGGAACTGAGGC-3′
rRNA18SRev	5′-CGGTCGGCATCGTTTATGGT-3′
ACLY For1-pRF/pHpRF	5′-AAGCTTGAATTCAGCCGATGGGGGCGGGGAAA-3′
ACLY Rev1-pRF/pHpRF	5′-GAATTCCATGGCTGCAGAGAGACCTGCTC-3′
ACLY For1-pGL3prom	5′-GAATTCAAGCTTAGCCGATGGGGGCGGGGAAA-3′
ACLY Rev1-pGL3prom	5′-GAATTCCATGGCTGCAGAGAGACCTGCTC-3′
CSFor	5′-GGCTTCCAAGGTGTACGACCCCGAG-3′
CSRev	5′-GGGCCCTTCTTAATGTTCTTAGCAT-3′
